# Coccolith dissolution within copepod guts affects fecal pellet density and sinking rate

**DOI:** 10.1038/s41598-018-28073-x

**Published:** 2018-06-27

**Authors:** Meredith M. White, Jesica D. Waller, Laura C. Lubelczyk, David T. Drapeau, Bruce C. Bowler, William M. Balch, David M. Fields

**Affiliations:** 1 Bigelow Laboratory for Ocean Sciences, 60 Bigelow Drive, East Boothbay, ME 04544 USA; 2 Present Address: Mook Sea Farm, 321 State Route 129, Walpole, ME 04573 USA

## Abstract

The most common biomineral produced in the contemporary ocean is calcium carbonate, including the polymorph calcite produced by coccolithophores. The surface waters of the ocean are supersaturated with respect to calcium carbonate. As a result, particulate inorganic carbon (PIC), such as calcite coccoliths, is not expected thermodynamically to dissolve in waters above the lysocline (~4500–6000 m). However, observations indicate that up to 60–80% of calcium carbonate is lost in the upper 500–1000 m of the ocean. This is hypothesized to occur in microenvironments with reduced saturation states, such as zooplankton guts. Using a new application of the highly precise ^14^C microdiffusion technique, we show that following a period of starvation, up to 38% of ingested calcite dissolves in copepod guts. After continued feeding, our data show the gut becomes increasingly buffered, which limits further dissolution; this has been termed the Tums hypothesis (after the drugstore remedy for stomach acid). As less calcite dissolves in the gut and is instead egested in fecal pellets, the fecal pellet sinking rates double, with corresponding increases in pellet density. Our results empirically demonstrate that zooplankton guts can facilitate calcite dissolution above the chemical lysocline, and that carbon export through fecal pellet production is variable, based on the feeding history of the copepod.

## Introduction

The conservative nature of calcium carbonate (CaCO_3_) in shallow ocean depths is a long-held standard of chemical oceanography. Shallow waters are supersaturated with respect to CaCO_3_ and dissolution is predicted only below the chemical lysocline, the depth representing a critical CaCO_3_ undersaturation, resulting in a distinct increase in CaCO_3_ dissolution^1^. However, evidence for dissolution above the lysocline has existed for some time^2–5^, with notable work from nearly two decades ago suggesting that as much as 60–80% of calcium carbonate may dissolve in the upper 500–1000 m of the ocean, well above the lysocline^2^. This phenomenon may be caused by multiple factors including, but not limited to, biologically-mediated dissolution, such as within aggregates of particulate organic matter experiencing high respiration rates^2^ or within zooplankton guts^2,6^.

*Calanus* spp. copepods have been shown to have gut pH levels as low as 5.4^7^, indicating they could provide microenvironments with conditions facilitating CaCO_3_ dissolution. One likely source of CaCO_3_ to dissolve in copepod guts is the calcite coccoliths of coccolithophores, a phytoplankton food source for copepods. However, previous experimental work estimated coccolith calcite dissolution within copepod guts at <8%^8^ and 73%^6^, perhaps due to major differences in experimental design, with the estimate of 8% dissolution resulting from comparisons of the morphology of coccoliths in fecal pellets to the morphology of coccoliths partially dissolved through dissolution experiments^8^, and the estimate of 73% resulting from chemical composition analyses of copepod fecal pellets and of the coccolithophore cells that the copepods were fed^6^. Modeling work indicates that 25% of calcite could be dissolved in copepod guts during alternating grazing and non-grazing periods, with less dissolution occurring during periods of constant grazing^9^.

## Results and Discussion

**Figure 1:**
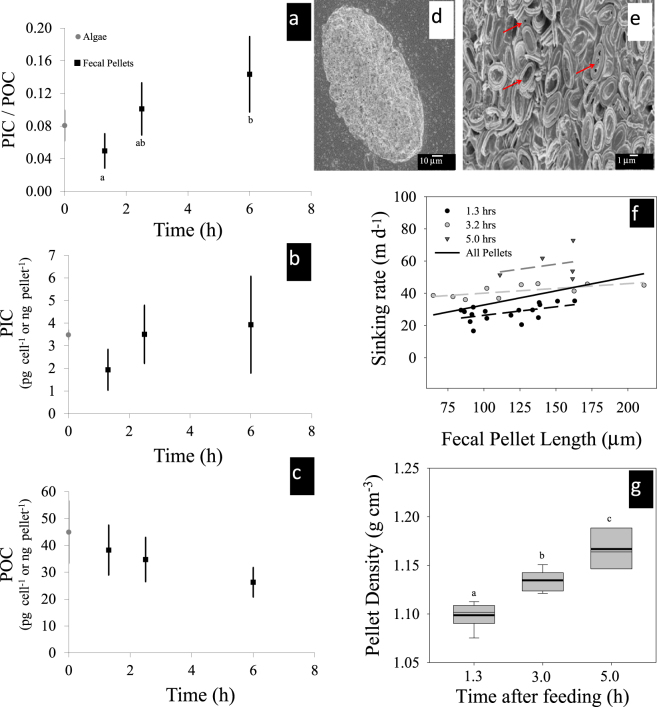
Dissolution of calcium carbonate in copepods’ guts. (**a**) Ratio of particulate inorganic carbon (PIC) to particulate organic carbon (POC) of algae (*Pleurochrysis carterae*) and of fecal pellets produced by *Acartia tonsa*. (**b)** PIC of algae and fecal pellets, presented as pg cell^−1^ or ng pellet^−1^, respectively. (**c)** POC of algae and fecal pellets, presented as ng pellet^−1^ or pg cell^−1^. Values in panels a–c are mean ± standard deviation. Algae PIC and POC were measured at the beginning of the grazing period. Copepods were allowed to graze for 1.3 hours, then removed from food. The PIC/POC of the first fecal pellet is not significantly lower than the PIC/POC of the algae (one-tailed *t*-test, *t* = 2.11, df = 6, *p* = 0.040). Different letters indicate a significant difference in the PIC/POC value of fecal pellets (one-way ANOVA, F = 8.51, df = 2, *p* = 0.01). No significant differences were found among fecal pellets with regard to PIC pellet^−1^ or POC pellet^−1^. Scanning electron micrographs of (**d)** an *A*. *tonsa* fecal pellet at 530X magnification and (**e)** of coccoliths within an *A*. *tonsa* fecal pellet at 5,060X magnification. These coccoliths show qualitative evidence of dissolution, as indicated by the red arrows. (**f)** Video-determined sinking rate of *A*. *tonsa* fecal pellets produced at different times post-feeding (**g)** Whisker plot showing calculated density of the pellets based on sinking rates using a modified Stokes flow equation^14^. Different letters indicate a significant difference in the calculated density fecal pellets (one-way ANOVA, F = 54.23, df = 2, *p* < 0.001).

Using a novel application of the ^14^C-microdiffusion technique^10,11^, we found an initial 38% dissolution of the coccolith calcite from *Pleurochrysis carterae* eaten by *Acartia tonsa* copepods, represented by a significant decrease in the ratio of particulate inorganic carbon to particulate organic carbon (PIC/POC) of initial fecal pellets, relative to the PIC/POC of the algae (*P*. *carterae*; Fig. 1a). The mean PIC/POC of the first fecal pellets produced by *A*. *tonsa* after feeding for 1.3 h following 24 h starvation was significantly lower than the PIC/POC of *P*. *carterae* (one-tailed *t*-test, *t* = 2.11, df = 6, *p* = 0.040), indicating initial dissolution of PIC consumed by the copepods (Fig. 1a). Fecal pellet PIC/POC increased in both the second and third set of fecal pellets produced, with the third set having significantly higher PIC/POC than the first set (one-way ANOVA, F = 8.51, df = 2, *p* = 0.01; Tukey’s HSD test, *p* = 0.009). The increase in the ratio is driven both by increasing PIC per pellet with time and decreasing POC per pellet with time (Fig. 1b,c). The increase in PIC per pellet with time indicates less dissolution occurs for coccoliths passing through the digestive tract, likely due to buffering of the digestive tract from the previously-dissolved calcite. The decrease in POC per pellet with time indicates more efficient digestion of organic carbon as the copepods’ guts are filled. Scanning electron microscope (SEM) micrographs show qualitative evidence of coccolith dissolution, mainly in the central area (Fig. 1d,e).

The first set of fecal pellets collected (~80 min post feeding) were produced prior to the typical gut passage time for *A*. *tonsa* (85–166 min^12^) and showed the lowest PIC/POC ratios of all pellets produced. The second set of pellets collected at 2.5 h past the start of the feeding period may represent a combination of pellets produced from digestion in a previously empty gut and pellets produced from digestion in an already-filled gut. The final set of pellets collected at 6 h past the start of the feeding period represents pellets produced from digestion in an already-full gut. Increasing PIC/POC in fecal pellets over time after the initiation of grazing supports modeling results indicating that dissolution of calcite in a copepod gut raises the gut saturation state, reducing further dissolution as grazing and digestion continues^9^. As predicted with models^9^, maximum calcite dissolution occurred during the early stages of feeding and as the gut remained full, dissolution slowed, or even ceased (Fig. 1a). Some copepods, including *Acartia* spp. undergo a diel feeding pattern creating alternate feeding and non-feeding periods^13^, meaning calcite dissolution in a copepod gut will be variable, depending on when the animal last fed.

Video-determined sinking rates of the three sets of fecal pellets produced and collected sequentially following an 80 min feeding period showed a doubling in the average sinking rate of fecal pellets collected 5 h after the start of the feeding period, relative to those collected 1.3 h after the start of the feeding period (Fig. 1f). This corresponds to the increase in PIC/POC of fecal pellets collected in a similar time frame from the ^14^C experiment (Fig. 1c). The density of each video-recorded sinking rate was calculated following a modified Stokes flow equation^14^ (Fig. 1g) and each subsequent set of fecal pellets had significantly different mean densities, with densities increasing with time since the feeding period started, again corresponding the increased PIC/POC found in the ^14^C experiment.

We have shown, through highly precise ^14^C tracing, that coccolith calcite dissolves in copepod guts. Dissolution is highest following non-feeding periods and dissolution decreases with time, likely as a result of the dissolved calcite buffering in the gut, a hypothesis that is supported with modeling work^9^. This represents a mechanism for up to 38% of ingested calcite to dissolve during the start of a feeding period, providing experimental evidence to support the hypothesis that biological processes can cause the dissolution of CaCO_3_ at depths above the chemical lysocline^2^. The consequence of the decrease in dissolution as the zooplankton guts are buffered is that fecal pellet density increases and sinking rates double, increasing the rate of export of carbon out of surface waters. Longer periods of feeding, pellet collection, and ^14^C analyses would further the empirical understanding of the dynamics governing rates of calcite dissolution in zooplankton guts.

## Methods

### Coccolithophore culture maintenance

*Pleurochrysis carterae* (CCMP 645) cultures were grown under axenic conditions in semi-continuous batch culture using L1-Si culture media^15^ prepared in filtered (0.2 *µ*m pore size), UV-sterilized, autoclaved seawater. Both the cultures and prepared media were continuously bubbled at approximately 500 mL min^−1^ with filtered (0.2 *µ*m pore size) compressed air. Cultures were maintained in an incubator set at 16.5 ± 0.5 °C with a light intensity of 470 *µ*mol photons m^−2^ s^−1^ photosynthetically active radiation (PAR) on a 14–10 h light-dark cycle. Daily measurements of cell density and *in vivo* fluorescence were made using a Moxi Z mini automated cell counter and a Turner 10-AU fluorometer, respectively. Based on these measurements, as soon as cultures neared the end of exponential phase, they were diluted with new, sterile L1-Si media to keep the cultures in exponential growth. Cultures were allowed to grow under these conditions for at least 9 generations before any experiments were performed.

### Copepod collection and maintenance

Zooplankton were collected via horizontal net (153 *µ*m) tows from a dock in East Boothbay, Maine, USA. Tow samples were immediately brought to the laboratory and adult *A*. *tonsa* were identified through a stereomicroscope and isolated into cultures maintained in an incubator at 16.0 ± 0.5 °C. Cultures were fed daily with an algal mixture of 60% *Rhodomonas marina* (CCMP 1319), 25% *Thalassiosira wiessflogii* (CCMP 1051) and 15% *P*. *carterae* (CCMP 645) to a final concentration of 20,000 cells mL^−1^. Only adult females were used for experiments and they were starved in filtered (0.2 *µ*m pore size) seawater for 24 h prior to experiments in order for their guts to clear of their previous diet.

### Coccolith dissolution experiment

**Figure 2:**
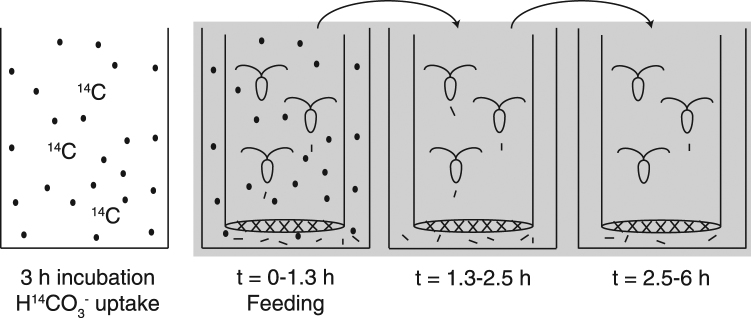
^14^C experimental schematic. The coccolithophore *Pleurochrysis carterae* was incubated with H^14^CO_3_^−^ for 3 h in the light in 20 mL glass scintillation vials. After the incubation, the vials were moved to the dark and a mesh-bottom tube containing 15 individual copepods (*Acartia tonsa*) was added to each vial and the copepods were allowed to graze on the algae for 1.3 h, producing fecal pellets. After the feeding period, the copepod tubes were moved through a series of six filtered seawater rinses and into a fourth vial containing non-radioactive filtered seawater and allowed to produce fecal pellets in that vial for 1.2 h before being moved through three filtered seawater rinses and into a final vial, where they remained until the end of the 6 h experiment. Each time the copepods were moved, the fecal pellets that they produced in that vial were collected for ^14^C analysis.

In order to utilize ^14^C as a tracer for organic and inorganic carbon moving through the copepods’ guts, we employed a novel application of the ^14^C-microdiffusion technique that is commonly used to determine algal photosynthetic and calcification rates^10,11^. The microdiffusion technique allowed for the independent measurement of ^14^C accumulated in both the POC and the PIC pool. This technique was preferred over the “differencing technique” where one subtracts the particulate organic carbon (POC) from the total carbon fixed. The concern with this technique (differencing technique) is that the calculated PIC comes from taking the difference between two big numbers (since calcification for most applications in the marine environment is only 1–5% of the total carbon fixed) which generates a large error value. Alternatively, the micro-diffusion technique provides a direct measure of the carbon which has been fixed via calcification into calcite. To collect these measurements a subsample of *P*. *carterae* culture was diluted to 60,000 cells mL^−1^, which is three times the optimal grazing density for *A*. *tonsa*. This subsample was split into three tissue culture flasks: one flask (Flask A, ^14^C-HCO_3_^−^-labeled algae) was immediately spiked with ^14^C-HCO_3_^−^ (3.7 × 10^7^ Bq per 180 mL algae); one flask (Flask B, ^14^C-HCO_3_^−^-labeled seawater) was spiked with ^14^C-HCO_3_^−^ after the 3 h incubation, just prior to adding copepods; and one flask (Flask C) was never spiked with ^14^C, so that it could be used for analyses which required non-radioactive material. After Flask A was spiked with ^14^C-HCO_3_^−^, a 35 mL subsample was removed and poisoned with 1 mL of buffered formalin to serve as a blank for algal uptake of ^14^C. Subsequently, Flasks A, B, and C were incubated for 3 h at 16.5 ± 0.5 °C and 415 *µ*mol photons m^−2^ s^−1^ PAR. A schematic of the experiment is provided in Fig. 2.

Following the 3 h incubation, the incubator light and room lights were turned off (except for a red overhead light to provide illumination for working), and the remainder of the experiment was performed in the dark in order to stop algal photosynthesis. From Flask A (^14^C-HCO_3_^−^-labeled algae) and the corresponding formalin blank, triplicate 1 mL subsamples were filtered onto 0.4 µm polycarbonate filters and their carbon was partitioned into organic and inorganic fractions by acidification and subsequent capture of ^14^CO_2_ (from particulate inorganic carbon, PIC) in a trap containing a Whatman GFA filter presoaked with 0.2 mL phenethylamine^11^. These samples allowed us to determine the radioactivity signal (disintegrations per minute, dpm) of organic and inorganic carbon assimilated by *P*. *carterae* during the 3 h incubation. In order to determine the relationship between the organic and inorganic ^14^C dpm signal to *µ*g organic and inorganic carbon, respectively, samples of cold algae from Flask C were taken for particulate organic carbon (POC) and PIC analyses. At the same time, the cell density of Flask C was measured with a Moxi Z mini automated cell counter and was assumed to be the same density as in the parallel ^14^C-HCO_3_^−^-spiked Flasks A and B. For POC analyses, 10 mL of cold algae were filtered onto triplicate pre-combusted Whatman GF/F filters. The filters were then fumed in 10% HCl to remove inorganic carbonates, dried, and subsequently analyzed on an ECS 4010 CHNSO Analyzer by Bigelow Analytical Services, providing a value of *µ*g organic C mL sample^−1^. For PIC analyses, 10 mL of cold algae were filtered onto 0.4 *µ*m polycarbonate filters in triplicate^16^. In order to remove calcium chloride from any remaining seawater, the filtered algae was rinsed with potassium borate buffer (with the pH adjusted to 8.0). The sample filters were stored in trace metal-free centrifuge tubes and later digested with 5 mL of 5% nitric acid. The calcium concentration of the resulting digest was a measured on a Jobin Yvon Ultima C inductively coupled-plasma atomic emission spectrometer to provide a value of *µ*g Ca mL sample^−1^. With the assumption that all calcium present represented calcium carbonate, this value was converted to *µ*g inorganic C concentration.

At the same time point, the darkened Flask B was spiked with ^14^C-HCO_3_^−^. By spiking Flask B with ^14^C-HCO_3_^−^ after the 3 h light incubation, but before adding copepods, we made the assumption that there is no uptake of ^14^C-HCO_3_^−^ by *P*. *carterae* in the dark, and this treatment served as a control for any dissolved ^14^C-HCO_3_^−^ that the copepods may take up from the seawater itself, as opposed to from the algae.

Adult female *A*. *tonsa* that had been starved for 24 h were moved to chambers with 5 mL internal volumes and with 200 *µ*m mesh screens on the bottom. This mesh size is large enough to allow fecal pellets to pass through, but small enough to retain adult *A*. *tonsa*. Each chamber was temporarily stored in a 20 mL scintillation vial containing 0.2 *µ*m filtered seawater. From each of Flask A and Flask B, 19 mL of algae was transferred to six replicate glass scintillation vials. The copepods in their mesh-bottomed chambers were immersed in the ^14^C-HCO_3_^−^-labeled algae or ^14^C-HCO_3_^−^-labeled seawater treatments with one chamber containing 15 copepods per replicate scintillation vial (Fig. 2). All vials were returned to the incubator with the lights turned off. The copepods were allowed to graze for 1.3 h, and every 15 min each mesh-bottomed chamber was slowly moved up and down in its scintillation vial in order to keep the algae suspended. After the 1.3 h grazing period, the copepod chambers were immersed in six separate non-radioactive filtered seawater rinses and finally immersed in new scintillation vials containing 19 mL of non-radioactive filtered seawater. At 2.5 h, the copepod chambers were moved to new scintillation vials containing 19 mL of non-radioactive filtered seawater and remained in those vials until the end of the experiment at 6 h (Fig. 2).

At 1.3, 2.5, and 6 h, the fecal pellets from each replicate (six replicates at t = 1.3 h; three replicates at t = 2.5 h and t = 6 h) were collected on 28 *µ*m mesh screens that allowed algal cells and coccoliths to pass through. The fecal pellets were then collected on 0.4 *µ*m polycarbonate filters and rinsed three times with filtered seawater, and subsequently counted to know how many pellets were on the filter. The filters with pellets were transferred to new scintillation vials and the carbon was partitioned into organic and inorganic fractions as was done for the algae previously.

The radioactivity of each sample fraction was measured on a Perkin Elmer Tri-Carb 3110TR scintillation counter and the total organic and inorganic carbon of the algae, fecal pellets, and copepods were calculated from the radioactivity of the organic carbon and inorganic carbon fractions. To determine *µ*g C dpm^−1^, we used the following equation:

1 $$\mu {\rm{g}}\,{\rm{C}}\,{{\rm{dpm}}}^{-1}=\frac{(\frac{\mu {\rm{g}}\,{\rm{C}}}{{\rm{mL}}\,{\rm{cold}}\,{\rm{algae}}})}{({{\rm{R}}}_{{\rm{s}}}-{{\rm{R}}}_{{\rm{f}}})}$$

where *µ*g C * (mL cold algae)^−1^ is determined from the POC or PIC analysis of the cold algae immediately following the 3 h incubation, R_s_ is the radioactivity (dpm mL^−1^) of the ^14^C-HCO_3_^−^-labeled algae, and R_f_ is the radioactivity (dpm mL^−1^) of the formalin blank. The calculation is the same for organic and inorganic carbon – the difference is using the *µ*g C mL^−1^ from either the POC or PIC analysis and the dpm value from either the organic fraction or the inorganic fraction, respectively. The algal cellular POC and PIC contents (pg C cell^−1^) were calculated using the results of the POC or PIC analysis (*µ*g C mL^−1^) and the cell density measured from the cold algae. Because *µ*g C mL^−1^ from the POC and PIC analysis represents the total (organic or inorganic) carbon per mL, the conversion to *µ*g C dpm^−1^ represents the total carbon in the fecal pellet, or copepod, not only the radio-labeled carbon. This was used to calculate the weight of organic and inorganic carbon in the fecal as follows:

2 $$\mu {\rm{g}}\,{\rm{C}}\,{{\rm{pellet}}}^{-1}=({{\rm{R}}}_{{\rm{p}}}-{{\rm{R}}}_{{\rm{b}}})\,\ast \,(\frac{\mu {\rm{g}}\,{\rm{C}}}{{\rm{dpm}}})$$

where R_p_ is the per pellet radioactivity (dpm pellet^−1^) of pellets produced by copepods that had been consuming ^14^C-HCO_3_^−^-labeled algae, and R_b_ is the per pellet radioactivity (dpm pellet^−1^) of pellets produced by copepods that had been in the ^14^C-HCO_3_^−^-labeled seawater treatment to account for any transfer of radioactivity from the seawater (as opposed to the ingested algae) to the fecal pellets.

Based on the calculations of organic and inorganic carbon content in the algae and fecal pellets, we calculated the particulate inorganic carbon to particulate organic carbon ratios (PIC/POC) of the algae and fecal pellets.

### Fecal pellet visual and sinking rate analyses

We performed an identical experiment to the one described above, but without ^14^C in order to collect fecal pellet samples for qualitative SEM imaging to look for evidence of dissolution of coccoliths and to determine the sinking rate of the fecal pellets. A subsample of our *P*. *carterae* culture was diluted to 60,000 cells mL^−1^ and aliquoted into glass scintillation vials (19 mL per vial). Three replicate vials were used to collect samples for SEM imaging and three separate replicate vials were used to collect samples for sinking rate analysis. Similar copepod chambers with a 200 *µ*m mesh bottom and 5 mL internal volume were placed with 15 adult female *A*. *tonsa* into the scintillation vials with algae. The copepods were allowed to graze for 1.3 h, then were moved to filtered seawater, and were moved again to new filtered seawater at 3.2 h. Fecal pellets were collected at 1.3, 3.0, and 5 h after the start of the grazing, which differs slightly from the timing of the ^14^C experiment. The entire experiment was carried out in the dark at 16.0 ± 0.5 °C.

For SEM imaging, fecal pellets were carefully transferred to a 0.4 µm polycarbonate filters already mounted with a carbon sticky tab on an SEM stub (one stub per replicate). Stubs were sputter-coated with gold using a Denton Desk IV sputter coater (Denton Vacuum, Moorestown, NJ, USA), and imaged on a Zeiss Supra25 field emission SEM (Carl Zeiss Microscopy, LLC, Thornwood, NY, USA).

For sinking rate analyses, fecal pellets were collected from each replicate at each timepoint and grouped by replicate/timepoint into 1 mL microcentrifuge tubes, which were refrigerated until analysis (<2 h). Sinking rates were measured in a 2 cm × 2 cm × 5 cm (L:W:H) corked cuvette with a small glass funnel (1 mm inner diameter) mounted in the center of the chamber. The tank and funnel were filled with 0.2 μm filtered seawater at a salinity of 32 and a water temperature of 20 °C (+/− 0.5 °C). Individual fecal pellets were gently transferred to the top of the funnel and allowed to passively sink through the funnel into the center of the cuvette. Sinking rates were measured from 3.0–3.5 mm from the top of the chamber. Filming was done with a Point Grey Flea 3 camera fitted with a 105 Nikon lens separated by a 25 mm bellow system to produce a field of view of 6 mm × 8 mm (W:H) for a resolution of 5.9 μm pixel^−1^. The vertical position of the pellet was recorded every 3–5 frames. Video images were recorded at 60 hz. The average velocity of the pellet was calculated from a minimum of 10 individual measurements.

Measured pellet size and sinking rates were used to calculate individual fecal pellet density from the Stokes flow equation modified to account for the cylinder shape^14^.

3 $$\omega =0.0790\frac{1}{\mu }({\rho }_{s}-\rho )g{L}^{2}{(\frac{L}{D})}^{-1.664}$$

where ω_s_ is the settling rate of the pellet,  μ and ρ are the viscosity and density of the water, g is acceleration due to gravity (9.81 m s^−1^) and L, D, and ρ_s_ are the length, diameter and density of the pellet.

### Statistical analyses

The PIC/POC ratio of *P*. *carterae* following the three hour incubation was compared to the PIC/POC ratio of the fecal pellets collected at 1.3 h using a one-tailed *t*-test. The PIC/POC ratios of the three sets of fecal pellets produced were compared using a one-way ANOVA with the time sampled as the grouping variable, followed by a Tukey’s honest significant difference test to determine which pellets were different from the others. Sinking rates were analyzed as a function of fecal pellet lengths by linear regression. Calculated densities were compared using a one-way ANOVA. Individual comparisons between treatments (time of production) were done using a Pairwise Multiple Comparison Procedures (Holm-Sidak method) in SigmaPlot 11.0.

### Data availability

Datasets from this work are available though the Biological and Chemical Oceanography Data Management Office (BCO-DMO) at http://www.bco-dmo.org/project/514415.
